# Comparison of self-efficacy among graduate teaching assistants before and after training

**DOI:** 10.1186/s12909-024-05431-0

**Published:** 2024-04-29

**Authors:** Hexiang Peng, Yiqun Wu, Ren Zhou, Jin Jiang, Xi Chen, Mengying Wang, Tao Ren, Chihui Yu, Tao Wu

**Affiliations:** 1 Department of Epidemiology and Biostatistics, School of Public Health, Peking University, Beijing, 100191 China; 2 Key Laboratory of Epidemiology of Major Diseases (Peking University), Ministry of Education, Beijing, 100191 China; 3 Key Laboratory of Carcinogenesis and Translational Research, Laboratory of Genetics, Peking University Cancer Hospital & Institute, Beijing, 100191 China; 4 School of Public Health, Peking University, Beijing, 100191 China; 5 Department of Equipment and Laboratory Management, Peking University Health Science Center, Beijing, 100191 China

**Keywords:** Medical education, Teaching assistants training, Self-efficacy

## Abstract

**Background:**

Teaching assistants (TAs) play a crucial role in pedagogical practices, and the TA training has emerged as a vital strategy for enhancing teaching quality and fostering effective interactions. The self-efficacy of TAs can substantially impact their performance. Nevertheless, little research has focused on the change in TAs’ self-efficacy following their training.

**Methods:**

A self-control quasi-experiment was conducted to examine shifts in the self-efficacy of Tas at Peking University before and after their TA training. A questionnaire was used to assess the change, and the reliability and validity of the questionnaire was also calculated. A paired data rank sum test was used to analysis the changes in TA self-efficacy before and after training.

**Results:**

A total of 372 TAs from School of Basic Medicine (*N* = 173), School of Pharmacy (*N* = 112), School of Public Health (*N* = 69), and other schools (*N* = 18) submitted complete questionnaires. The questionnaire showed a good performance in internal reliability and validity test (Cronbach’s alpha index = 0.906, and KMO value was 0.903). Participants had a median total self-efficacy score of 88 and 85 before and after the TA training, respectively, which shows a lack in the total TA self-efficacy score following the TA training (*P* < 0.001). TAs who have no desire to becoming a college instructor have a higher self-efficacy when compared to TAs who have expressed neutral attitudes in becoming college instructors.

**Conclusion:**

The participated TAs display a lack of self-efficacy after attending the TA training at Peking University. Therefore, it is necessary to establish and strengthen TA’s self-efficacy beyond academic skills when designing and delivering TA training programs at Peking University.

**Supplementary Information:**

The online version contains supplementary material available at 10.1186/s12909-024-05431-0.

## Background

An effective Teaching Assistant (TA) serves as a vital link connecting educators and students, playing a pivotal role in conveying information within medical schools. This role offers graduate students the chance to collaborate closely with faculty members and gain firsthand experience in teaching responsibilities. Nevertheless, it is imperative that TAs receive proper training in practical teaching skills to fulfill these demands [1]. Thus, comprehensive TA training holds great importance for students, graduate students, and educators alike.

The TA training system, dating back to the 1900s in North America, was established to enhance teaching effectiveness, refine the graduate education system, and cultivate a high-quality talent pool for future teaching roles [2]. In medical schools, TA training programs primarily focus on improving teaching skills and promoting their understanding of the fundamental concepts of medical education, instilling a foundation for future career development. A TA training program could improve the overall capacity of teaching assistants, to further promote teaching effectiveness. It is worth highlighting that the psychological well-being of TAs, such as their self-efficacy, can significantly impact their teaching effectiveness through various channels. In 1977, Albert Bandura first proposed self-efficacy theory, which proposed the concept of self-efficacy as “how well one can execute courses of action required to deal with prospective situations” [3, 4]. In brief, self-efficacy is a person’s perceived capabilities to learn or perform actions to succeed in a particular situation [5]. Bandura’s self-efficacy theory summarized four different sources of self-efficacy: performance accomplishments, vicarious experiences, verbal persuasion, and physiological feedback [3]. Widely explored in the field of education, previous studies have concluded that teacher self-efficacy plays a crucial role in influencing student achievement, motivation, teacher performance, and commitment [6–8]. Both self-efficacy and teaching approach have significant impacts on teaching performance and student learning, especially in the absence of practiced skills or developed knowledge. It’s important to note that while they contribute synergistically, they represent distinct aspects of TA training [6–8]. Thus, TA self-efficacy should be an important index to evaluate how TA training programs pertain to specific goals. TA self-efficacy refers to TAs’ belief in their teaching skills and the ability to complete teaching tasks [6, 8], which plays a pivotal role in shaping teaching approaches, with exhibiting greater confidence in their teaching abilities often adopting a more student-centered rather than teacher-centered approach to instruction [7]. Additionally, teachers’ self-efficacy may be altered by many factors, such as teachers’ intrinsic personal traits (experience, flexibility, hard work, perseverance, motivation, attitude, resourcefulness, and how they see themselves as teachers), extrinsic educational conditions, pre-service teacher education, and in-service teacher training [9, 10]. Therefore, TA training might play a crucial role in improving their self-efficacy. However, while Japanese universities have recognized the psychological significance of TA training and have incorporated corresponding psychological training components, there is still a lack of attention to TAs’ self-efficacy in other countries, especially in Asia [11].

Consequently, the current evaluation of TA training mainly focuses on improving teaching skills and gathering students’ feedback, with minimal regard to the psychological activities of TAs themselves [12, 13]. While some universities have strengthened the psychological development of TAs, the primary focus has been on teaching ethics and education beliefs among faculty [14, 15], often overlooking the pivotal element of TAs’ self-efficacy. In addition, the impact of TA training on TA’s self-efficacy has gone largely unnoticed. It’s important to note that the majority of studies in this domain have been conducted in western countries, typically with relatively small sample sizes [16–18]. A previous study explored the influences of training on TAs’ self-efficacy among secondary school TAs, which found TA self-efficacy might be influenced by Bandura’s four sources of information, outcome expectations, and whole school support and norms [19]. Notably, the parameters of training, iterative process of training, and involvement in the process were also important characteristics related to TAs’ self-efficacy [19]. Additionally, the nature and subthemes of TA training programs vary significantly across different studies. Therefore, there is a pressing need for further research to delve into training influence in TA’s self-efficacy and to enhance the overall effectiveness of TA training.

Peking University Health Science Center has a complete TA training system since 2015, which aims to help TAs understand their future work, shape teaching concepts, and enhance work confidence, to quickly take up their post and then improve teaching effectiveness. However, the influence of TA training on TAs’ self-efficacy has not been evaluated and reported. Therefore, we aim to investigate the TAs’ self-efficacy changes before and after TA training and search for potential factors influencing graduates’ TAs’ self-efficacy in Peking University.

## Methods

A before-and-after self-control study design was implemented, assessing the impact of Peking University Health Science Center’s TA training program. The study involved an examination of TA self-efficacy, conducted during the fall semesters from 2017 to 2019 at Peking University Health Science Center. Graduate students were mandated to participate in the training program and obtain certification before commencing their roles as a TA.

### Participants

The participants were medical graduate students who had participated in TA training at Peking University Health Science Center from 2017 to 2019. Only TAs who completed the questionnaires before and after training were included. Participants were excluded if they missed any surveys or if the questionnaires were answered incompletely. Finally, 372 TAs who participated in the pre-training and post-training questionnaires were included in the analysis. All participants provided informed consent.

### TA training

Peking University Health Science Center has a routine TA training program, which aims to help TAs understand the job requirements, shape teaching concepts, and enhance their teaching capacity. The pre-job training program included a series of training activities, including TA workshops, TA salons, and demonstration lectures (See Table 1). These activities were delivered as both compulsory and elective courses/lectures. Each TA was required to obtain three credits of compulsory courses/lectures and a minimum of two credits from elective courses/lectures. Each course or lecture had a duration of at least two hours. The courses/lectures mainly introduced educational principles, teaching skills, and the integration of modern educational technology into instructional practices. Specific elective courses/lectures were tailored to address distinct teaching skills required for theoretical and laboratory courses. Upon successful completion of all the mandatory courses, trainees were expected to submit a report providing feedback on their TA training experience. To obtain TA certification (see Table 1), trainees were required to accumulate a total of six credits during the TA training program and pass the TA training assessment.

### Data collection

Data were systematically collected before and after TA training during the fall semesters across three consecutive years from 2017 to 2019. Data collection incorporated both quantitative and qualitative data. Prior to the training, TAs were administered to complete a self-efficacy questionnaire. Upon completion of the pre-job training requirements, they were invited to submit a follow-up questionnaire and, receive their teaching assistant certification. The questionnaire was initially designed by experts from the Peking University Medical Education Development Center and the research team. Furthermore, the reliability of the questionnaire items was evaluated using Cronbach’s alpha as an indicator of internal consistency, and the validity was assessed through factor analysis, with a KMO index exceeding 0.8 serving as the eligibility threshold. The TA self-efficacy survey comprised ten questions, as detailed in Table 2. Each question was scored on a scale of 0-100, and an average score across all ten questions was computed to determine the TA self-efficacy level. The questionnaire also encompassed other related indicators, which were outlined in Table 2. In addition to quantitative data, qualitative data were collected to interpret the relationship between self-efficacy and TA training. The qualitative survey questions aimed to uncover one’s belief on one’s strengths and concerns associated with being a TA, as well as to investigate the advantages and disadvantages of taking the TA position.

### Statistical analysis

Median and quartile were used to describe the distribution of TAs’ self-efficacy scores before and after training. A rank sum test of paired data was used to compare their self-efficacy scores before and after training. Similar analyses were also performed among different subgroups. The linear regression model was used to evaluate the potential influence factor of self-efficacy levels, with the difference in total self-efficacy score as the dependent variable. All statistical analysis was performed using SPSS software. *P* < 0.05 was considered statistically significant.

**Table 1:** Overall training arrangement for graduate teaching assistants of Peking University Health Science Center

Courses	Training arrangement	Training topics	Training form	Credits
Compulsory courses	Opening ceremony	The significance of teaching assistant’s work and the previous management system	Group teaching	1 credit
		Introduction of teaching assistant training arrangement		
		Introduction to graduate teaching assistant experience		
		What makes a good teaching assistant		
	Online courses	1.Teaching competence development in graduate education	Choose one topic and finish online learning, offline submission of learning feedback	1 credit
		2. A century’s reflection on medical education		
		3. Characteristics of medical undergraduates and communication skills between teachers and students		
	TA salon	How to be a good teaching assistant (Theoretical courses/Experimental courses)	Solon	1 credit
Elective courses	Teaching skills	Teaching lecture	Group teaching	1 credit for each lecture trainees were required to select any 2 of them and obtain 2 credits
		Teaching salon	Solon	
		Demonstration lecture	Group teaching, interaction	
Final assessment	Feedback	Submission of learning feedback for the training	Report	1 credit

**Table 2:** Questions for self-efficacy survey before and after the teaching assistants training

Numbers	Questions
Q1	Can you impart new knowledge accurately to students?
Q2	Can you facilitate class group discussion?
Q3	Can you create conditions to help students actively participate in learning activities?
Q4	Can you adjust your teaching activities according to students’ learning characteristics?
Q5	Can you design homework for students?
Q6	Can you fairly grade students’ performance and assignments?
Q7	Can you provide constructive feedback or opinions to students?
Q8	Can you solve some conflicts among students (such as cheating, plagiarism, etc.)?
Q9	Do you have confidence that you will be popular with students?
Q10	Can you have confidence that you will be worthy of the name of teacher among students?
Other questions	Have you ever served as a teaching assistant this semester? Have you ever been interested in becoming a university teacher? Do you think training is helpful to improve your self-efficacy?
Qualitative questions	What are your concerns and strengths as a teaching assistant
	What do you think are the advantages and disadvantages of working as a teaching assistant

## Results

A total of 372 TAs completed questionnaires both before and after their TA training during the fall semesters spanning from 2017 to 2019, with participant numbers distributed as follows: 107 in 2017, 130 in 2018, and 135 in 2019. Nearly half of these graduate TAs were from the School of Basic Medicine. Among the participants, 174 (46.8%) were scheduled to begin their TA term in the current semester, and 237 (63.7%) expressed an interest in pursuing careers as college instructors. Before embarking on the TA training, almost three-quarters of the participants believed that such training could enhance their teaching self-efficacy, while only 21 (5.6%) students held the view that it might not be helpful.

The results of reliability and validity analyses demonstrated robust internal consistency for the questionnaire (Cronbach’s alpha index = 0.906), and after removing individual items, the Cronbach’s alpha index consistently exceeded 0.85. The questionnaire also performed well in the validity test, with a KMO (Kaiser-Meyer-Olkin) value of 0.903, and a p-value for Bartlett’s test of sphericity less than 0.05. The two-factor solution provided a total explained variance of 65.57% (Supplementary Table 1). Supplementary Table 2 presents the factor loadings (after rotation). All items had a factor loading above 0.60 in the assigned factor. These findings confirm the reliability and validity of the survey, underscoring the questionnaire’s quality.

### Changes in TA self-efficacy

As shown in Table 3, the TA had a median total self-efficacy score of 88 before the TA training and 85 after the TA training (*P* < 0.001). However, the self-efficacy change was no longer significant in Q6 (fair grading) and Q10 (fulfill teacher responsibilities). In addition, Tas showed a lack of self-efficacy on the other eight aspects (*P* < 0.001, Table 3).

### TA self-efficacy changes in different subgroups

After training, the TA self-efficacy scores were significantly lower than before training, similar across all three years from 2017 to 2019 (See Table 4). Significant differences were observed among different colleges (*P* = 0.048). The TAs’ self-efficacy in the School of Basic Science and the School of Pharmacy was significantly lower after training (*P* < 0.001). However, teaching experience, motivation to become a college instructor, and belief about improving TAs’ self-efficacy after TA training were not statistically associated with the self-efficacy score differences before and after training.

### Factors influencing self-efficacy changes

In the multivariate analysis, no significant association was observed for the change of total self-efficacy level (Table 5). However, those who were disinterested in becoming a college teacher were significantly associated with an increased self-efficacy level compared with neutral TAs (β:6.89, 95%CI: 0.94,12.83). TAs from School of Pharmacy tended to gain higher self-efficacy level in facilitating class group discussion (β:5.83, 95%CI: 1.46,10.21) and creating conditions to help students actively participate in learning activities (β:5.05, 95%CI: 0.52,9.58) (Supplementary Tables 1 and 2).

**Table 3:** Self-efficacy levels of 372 teaching assistants before and after training in Peking University Health Science Center from 2017 to 2019

Questions	Before the training	After the training	Changes	*P*-value
	Median (Quartile interval)	Median (Quartile interval)	Median (Quartile interval)	
Q1	90 (80, 90)	83 (80, 90)	-3 (-10, 4)	< 0.001 *
Q2	90 (80, 90)	82 (76, 90)	-3 (-12, 4)	< 0.001 *
Q3	90 (80, 95)	84 (75, 91)	-3 (-13, 4)	< 0.001 *
Q4	88 (80, 90)	81 (74, 90)	-3 (-12, 3)	< 0.001 *
Q5	90 (80, 90)	82 (74, 91)	-2 (-12, 5)	< 0.001 *
Q6	95 (90, 100)	95 (88, 100)	0 (-5, 5)	0.406
Q7	90 (80, 95)	86 (80, 94)	-1 (-10, 4)	< 0.001 *
Q8	90 (80, 95)	84 (75, 91)	-3 (-11, 4)	< 0.001 *
Q9	90 (80, 95)	86 (79, 93)	-1 (-9, 4)	< 0.001 *
Q10	90 (85, 100)	90 (84, 100)	0 (-5, 5)	0.364
Total score	88 (83, 93)	85 (79, 91)	-3 (-9, 2)	< 0.001 *

*: statistic significant

**Table 4:** Self-efficacy of 372 teaching assistants before and after training in different subgroups in Peking University Health Science Center from 2017 to 2019

Variables		Before training	After training	Changes	*P* value^a^	*P * value^b^
		Median (Quartile interval)	Median (Quartile interval)	Median (Quartile interval)		
**Total score(***N* **=** **372)**		88 (83, 93)	85 (79, 91)	-3 (-9, 2)	< 0.001*	
**Year**						0.072
2017 (*n* = 107)		90 (85, 93)	86 (81, 92)	-2 (-6, 2)	0.006*	
2018 (*n* = 130)		87 (82, 92)	82 (76, 90)	-4 (-11, 2)	< 0.001*	
2019 (*n* = 135)		89 (83, 94)	86 (79, 91)	-1 (-9, 3)	0.006*	
**Schools**						0.048*
School of Basic Medicine (*n* = 173)		88 (84, 92)	84 (78, 90)	-4 (-9, 1.5)	< 0.001 *	
School of Pharmacy(*n* = 112)		88 (83, 94)	86 (78, 91)	-4 (-11, 2)	< 0.001 *	
School of Public Health (*n* = 69)		89 (82, 94)	87 (81, 94)	-1 (-5, 4)	0.168	
Other schools (*n* = 18)		91 (83, 96)	87 (82, 93)	-1 (-5, 3)	0.166	
**Have you served as a TA this semester?**						0.545
	No (*n* = 198)	88 (83, 93)	88 (84, 92)	-3 (-9, 3)	< 0.001*	
	Yes (*n* = 174)	85 (80, 91)	84 (79, 91)	-4 (-9, 2)	< 0.001*	
**Want to be a college teacher?**						0.402
	No (*n* = 19)	87 (85, 94)	82 (78, 92)	-5 (-12, -1)	< 0.001*	
	Neutral (*n* = 116)	87 (82, 92)	86 (80, 90)	-2 (-6, 2)	0.001*	
	Yes (*n* = 237)	89 (84, 93)	85 (79, 91)	-3 (-9, 2)	< 0.001*	
**Do training improve your self-efficacy?**						0.354
	No (*n* = 21)	86 (82, 95)	87 (81, 95)	-2 (-10, 5)	0.412	
	Yes (*n* = 266)	89 (84, 93)	86 (80, 92)	-3 (-8, 2)	< 0.001*	
	Unsure (*n* = 85)	87 (82, 91)	82 (78, 88)	-4 (-10, 1)	< 0.001*	

a: Test P value of self-efficacy score difference before and after training

b: Test P value of self-efficacy score difference in different subgroups before and after training

**Table 5:** Factors influencing the total self-efficacy level before and after training

Variables		β(95CI%)	*P*
Year			
2017 (*N* = 107)		Reference	
2018 (*N* = 130)		-2.12 (-4.78,0.53))	0.262
2019 (*N* = 135)		-0.51 (-3.11,2.10)	0.395
Schools			
Other schools (*N* = 18)		Reference	
School of Basic Medicine (*N* = 173)		-1.00 (-3.55,1.55)	0.193
School of Pharmacy (*N* = 112)		2.73 (-0.06,5.52)	0.273
School of Public Health (*N* = 69)		3.23 (-1.67,8.13)	0.502
Have you served as a TA this semester?			
No (*N* = 198)		Reference	
Yes (*N* = 174)		0.50 (-1.71,2.71)	0.443
Are you attending your first TA training?			
No (*N* = 9)		Reference	
Yes (*N* = 363)		0.32 (-6.23,6.88)	0.580
Want to be a college teacher?			
Neutral (*N* = 116)		Reference	
No (*N* = 19)		2.75 (-2.04,7.54)	0.117
Yes (*N* = 237)		1.00 (-3.67,5.66)	0.170
Dose training improve your self-efficacy?			
No (*N* = 21)		Reference	
Unsure (*N* = 85)		-1.11 (-5.58,3.35)	0.681
Yes (*N* = 266)		-2.49 (-7.21,2.23)	0.890

## Discussion

The study centered on examining alterations in self-efficacy following TA training within the cohort of graduate TAs. The outcomes indicated that the existing TA training program at Peking University was ineffective in enhancing the teaching self-efficacy of TAs.

The TA’s self-efficacy plays a critical role in the teaching effect and can profoundly impact their future career development [20]. In fact, most of the previous studies focused on the influence of TA training on the teaching effect but paid little attention to the TAs’ self-efficacy [21], while most of those involving graduate students often reported an increase in self-efficacy following TA training [22–25]. A study from Brandeis University reported that TAs could better understand the challenges they could encounter in future teaching roles [26]. However, our study observed a lack of self-efficacy improvement among TAs after completing their training. The inconsistency in findings can be attributed to several factors, including variations in the design and/or the major goal of TA training programs, the specific focus of training activities, the skill requirements for TAs in different courses, disparities in laboratory proficiency, and other related aspects. Another critical reason contributing to the decline in self-efficacy might result from TAs initially underestimating the requirements of the TA position before training. TA training serves as a critical means for TAs to comprehend the teaching objectives and fulfill the fundamental requirements for the positions. After training, TAs may re-evaluate the gap between their abilities and the demands of the job, leading to a decline in their self-efficacy. The qualitative survey responses from TAs in our study revealed significant concerns and challenges they might face in their TA positions. These concerns encompassed limited professional knowledge to address students’ inquiries, poor communication skills, insufficient experimental and theoretical knowledge, difficulty in handling teaching-related stress, managing unexpected or emergent situations, time constraints, and establishing authorities as TAs. Therefore, those apprehensions might intensify and become more explicit following TA training, subsequently leading to a reduction in their self-efficacy.

Prior experience has been regarded as a critical factor and was shown to significantly improve TA self-efficacy [27]. Moreover, TAs’ teaching experience could improve their methodological research skills [28]. Although previous studies have reported that TAs with more extensive experience tend to perform better in teaching [29], no specific evidence has been reported on TAs’ self-efficacy. Our study did not find a positive association between TA experience and TAs’ self-efficacy. Some reasons might explain this. Different courses, particularly those involving laboratory work, may impose varying standards on TAs, necessitating a greater depth of knowledge and preparation to navigate these challenges. Bran stetter et al. reported that most TAs deemed it unethical to teach as a TA without adequate preparation [14], which may help to explain the decline of the self-efficacy level after training while a certain amount of the trainees might find they were not fully ready for the job. Additionally, we did not find associations between the motivation to become a university teacher and TAs’ self-efficacy. However, concerning the accurate transmission of new knowledge to students, those who expressed no desire to pursue a college teaching career exhibited higher self-efficacy compared to TAs with neutral aspirations. One plausible explanation is that TAs uninterested in becoming college teachers may have initially had lower expectations, while those who aspired to become teachers possessed higher levels of professional knowledge and expectations. Limited evidence was available on the association between professional development motivation and self-efficacy. Further studies are needed to explore the relationship to provide insight into the improvement of TA training programs.

Our survey possesses several notable strengths. Firstly, the study was the largest scale study concentrating on the changes in TAs’ self-efficacy following TA training in medical schools. Benefiting from the comprehensive TA training program at Peking University, we were able to acquire a large sample size of participants and collect data of high quality. Secondly, our study represented the first instance of demonstrating the alteration in TAs’ self-efficacy before and after TA training in Chinese medical schools. While previous research has focused on developing TA reflection systems to improve self-efficacy [30], there has been limited focus on the Chinese higher educational settings. Our study brought new insights into the improvement of TA training program. Subsequent research endeavors are warranted to explore the underlying factors of this phenomenon, with the aim of refining the TA training program.

There are several limitations. Firstly, all participants were recruited from schools in Peking University Health Science Center, and the results may not be generalized to other schools. Secondly, due to the lack of demographic characteristics of the participants, such as gender and age, stratified analysis and multivariate analysis were unable to be performed in the current study. However, the TAs were all first- or second- year graduate students, and there was not much difference in the age of graduate students. Additionally, we conducted multivariate analysis adjusting for calendar year, schools, and other potential covariates to investigate the potential factors for the change of self-efficacy levels. Due to incomplete collection of some characteristic variables, only several important potential confounders were adjusted in the analysis. This might lead to confounding bias. Therefore, our study provides a rough clue for the follow-up teaching assistant training. Also, the training program might lack of efficacy in improving their confidence and a more comprehensive training program should be arranged. Moreover, we acknowledged the shortcomings of the scale as well as our training programs: our scale was not the professional scale of self-efficacy assessment in the previous studies, such as the teaching self-efficacy measurements summarized by Moran et al. [31]. There were many measurements of teaching self-efficacy with different items and characteristics [31]. Furthermore, we acknowledge that wherein certain questions in our questionnaire were found to be ambiguous and exhibited overlap in the domains of self-esteem, self-efficacy, and self-concept. Defined as the extent of an “individual’s perception of his or her ability to perform a specific behavior“ [32], self-efficacy pertains to the judgments individuals make about their competence in executing a task within a specific context. This judgment may influence or regulate the intention to initiate, display, or resist certain behaviors. Additionally, in line with Rosenberg’s definition [33], self-esteem involves the assessments and evaluations we make about our self-concept. Self-concept encompasses the overall idea of who a person believes they are dynamic construct shaped by individual perceptions of the personal self, capable of guiding behavior towards the fulfillment of needs[35]. Despite these nuanced distinctions, the differences between the three concepts were not always apparent, leading to numerous similarities and even overlaps[33–35], like Q9 and Q10. However, all Cronbach’s coefficients after deleting each item were greater than 0.89, which implied that removal of any question might not improve the validity of the questionnaire. Future studies should update the TA training program and the evaluation questionnaire in the future TA training to focus more on self-efficacy. Our questionnaire was developed according to the characteristics of our training and the focus content, which may need to be strengthened for further extension. In addition, our training program provided more on pre-preparation and practices of basic skills, ideas, and psychological qualities for the teaching assistants who were about to take up their positions, rather than specifically designed to improve their self-efficacy. Therefore, the results of the current study only showed that the current training program needs to be strengthened in improving the self-efficacy of teaching assistants and cannot be extended to general TA training. Finally, due to the self-controlled design of this study, it is difficult to avoid the bias caused by the time trend. Therefore, the interpretation of the results needs to be cautious.

## Conclusion

The findings suggested that the existing Teaching Assistant training program at Peking University might not improve the self-efficacy of teaching assistants. Consequently, beyond the focus on teaching skills training, it is imperative to incorporate elements that build and fortify the self-efficacy of TAs when revamping TA training programs at Peking University.

### Electronic supplementary material

Below is the link to the electronic supplementary material.

Supplementary Material 1

## Data Availability

The datasets generated and/or analyzed during the current study are not publicly available due to confidentiality agreement but are available from the corresponding author on reasonable request.

## References

[1] Holmes NG, Martinuk MS, Ives J, Warren M (2013). Teaching Assistant Professional Development by and for TAs. PHYS TEACH.

[2] Stewart RA, Rueter T (1990). Communication Educ.

[3] Bandura A (1977). Self-efficacy: toward a unifying theory of behavioral change. PSYCHOL REV.

[4] Vaughan-Johnston TI, Jacobson JA. Self-efficacy Theory. 2020:375–379.

[5] Lopez-Garrido G. Bandura’s Self-Efficacy Theory Of Motivation In Psychology. 2023.

[6] Bourne MJ, Smeltzer SC, Kelly MM (2021). Clinical teacher self-efficacy: a concept analysis. Nurse Educ Pract.

[7] Smith CR, Menon D, Wierzbicki A, Dauer JM (2023). Exploring STEM teaching assistants’ self-efficacy and its relation to approaches to teaching. CBE Life Sci Educ.

[8] Smith CR, Delgado C (2021). Developing a model of Graduate Teaching Assistant Teacher Efficacy: how do high and low teacher efficacy teaching assistants compare?. CBE Life Sci Educ.

[9] Wray E, Sharma U, Subban P (2022). Factors influencing teacher self-efficacy for inclusive education: a systematic literature review. Teach Teach Educ.

[10] Mehmood N. Factors impacting EFL teachers’ Self-efficacy: a theoritical perspective. Engl Lang Teach. 2019;12(4):39–48.

[11] Feng F, Fan Y (2019). Comparative research on Postaraduate Teaching Assistant Cu tivation and manacement between China and Us一Harvaro University and Peking University as an Example. Peking Educ.

[12] Barr M, Wright P (2019). Training graduate teaching assistants: what can the discipline offer?. Eur Polit Sci.

[13] Blatchford P, Russell A, Bassett P, Brown P, Martin C (2007). The role and effects of teaching assistants in English primary schools (years 4 to 6) 2000–2003. Results from the class size and pupil-adult ratios (CSPAR) KS2 Project. Brit Educ Res J.

[14] Branstetter SA, Handelsman MM (2000). Graduate teaching assistants: ethical training, beliefs, and practices. Ethics Behav.

[15] Brown CE (2016). Ethical issues when Graduate Students Act as mentors. Ethics Behav.

[16] Chiu PHP, Corrigan P. A study of graduate teaching assistants' self-efficacy in teaching: fits and starts in the first triennium of teaching. Cogent Educ 2019;6(1):1579964.

[17] Andrew K, Richards R, Levesque-Bristol C (2014). Developing teaching Assistant Self-Efficacy through a Presemester Teaching Assistant Orientation. Res Q Exerc Sport.

[18] Prieto LR, Meyers SA (1999). Effects of training and supervision on the self-efficacy of psychology graduate teaching assistants. Teach Psychol.

[19] Wheeler LB, Maeng JL, Chiu JL, Bell RL (2017). Do Teaching assistants Matter? Investigating relationships between Teaching assistants and Student outcomes in Undergraduate Science Laboratory classes. J Res Sci Teach.

[20] Alicea-Munoz E, Espar Masip J, Sullivan CS, Schatz MF. Assessing a GTA professional development program. *2017 Physics education research conference.* 2017:32–35.

[21] Rosse-Richards KA, Velasquez JD, Nelson DB, Levesque-Bristol C. *The Influence of a Teaching Assistant Orientation on Teaching Assistant Perceptions of Self-Efficacy.* 2013.

[22] Salloum M. *Training Effective and Confident Computer Science TAs.* 2020.

[23] Haque A, Meadows KN. Impact of the Lead TA Program on the perceived disciplinary instructional competence of Graduate Teaching assistants. Can J Scholarsh Teach Learn 2020, 11(2).

[24] Pelton JA (2014). Assessing Graduate Teacher Training Programs: can a teaching seminar reduce anxiety and increase confidence?. Teach Sociol.

[25] Romm I, Gordon-Messer S, Kosinski-Collins M (2010). Educating young educators: a pedagogical internship for undergraduate teaching assistants. CBE Life Sci Educ.

[26] Prieto LR, Altmaier EM (1994). The relationship of prior training and previous teaching experience to self-efficacy among graduate teaching assistants. Res High Educ.

[27] Feldon DF, Peugh J, Timmerman BE, Maher MA, Hurst M, Strickland D, Gilmore JA, Stiegelmeyer C (2011). Graduate Students’ teaching experiences improve their Methodological Research skills. Science.

[28] Mewis K, Dee J, Lam V, Obradovich S, Cassidy A (2018). A New Self-Assessment Teaching Assistant Survey for Growth and Development. Teach Learn inquiry-the ISSOTL J.

[29] Terui Y, Imamura R, Egi H. Development of a Teaching Assistant Reflection System to Improve Self-Efficacy. *Proceedings OF* 2020 *IEEE International conference on teaching, assessment, and learning for engineering (IEEE TALE 2020).* 2020:652–657.

[30] Tschannen-Moran M, Hoy AW, Hoy WK (1998). Teacher efficacy: its meaning and measure. REV EDUC RES.

[31] Bandura A (1997). The anatomy of stages of change. AM J Health Promot.

[32] Rosenberg M, Schooler C, Schoenbach C, Rosenberg F (1995). Global self-esteem and specific self-esteem - different concepts, different outcomes. Am Sociol Rev.

[33] Van Gurp S (2001). Self-concept of deaf secondary school students in different educational settings. J Deaf Stud Deaf Educ.

