# Supplementary material for: Comparison of self-efficacy among graduate teaching assistants before and after training
Source: BMC Med Educ. 2024 Apr 29;24:471. doi: 10.1186/s12909-024-05431-0 (PMC11059735; doi:10.1186/s12909-024-05431-0)
Supplement: Supplementary file 1 — Supplementary Material 1 [file 12909_2024_5431_MOESM1_ESM.docx]

**Supplementary tables**

| **Supplementary table 1 Total explained variance of the Initial Eigenvalues** | | | |
| --- | --- | --- | --- |
| **Components** | **Total** | **Variance (%)** | **Cumulative (%)** |
| 1 | 5.50 | 55.03 | 55.03 |
| 2 | 1.05 | 10.54 | 65.57 |
| 3 | 0.79 | 7.90 | 73.47 |
| 4 | 0.60 | 5.98 | 79.45 |
| 5 | 0.54 | 5.36 | 84.81 |
| 6 | 0.39 | 3.89 | 88.70 |
| 7 | 0.34 | 3.39 | 92.09 |
| 8 | 0.32 | 3.18 | 95.27 |
| 9 | 0.25 | 2.54 | 97.81 |
| 10 | 0.22 | 2.20 | 100.00 |

**Supplementary table 2. Factorial loads (after rotation) for the questionnaire.**

| **Subscales** | **Factor 1** | **Factor 2** |
| --- | --- | --- |
| Q1 | 0.819 | -0.251 |
| Q2 | 0.797 | -0.418 |
| Q3 | 0.708 | -0.434 |
| Q4 | 0.824 | -0.188 |
| Q5 | 0.696 | 0.225 |
| Q6 | 0.646 | 0.426 |
| Q7 | 0.741 | 0.283 |
| Q8 | 0.711 | 0.228 |
| Q9 | 0.826 | -0.108 |
| Q10 | 0.615 | 0.466 |

**Supplementary table 3 Multivariable-adjusted estimates for confidence level of top five question before and after training**

| **Variables** | | **β(95CI%) for Q1** | **β(95CI%) for Q2** | **β(95CI%) for Q3** | **β(95CI%) for Q4** | **β(95CI%) for Q5** |
| --- | --- | --- | --- | --- | --- | --- |
| Year |  |  |  |  |  |  |
|  | 2017 (N=107) | Reference | Reference | Reference | Reference | Reference |
|  | 2018 (N=130) | -3.10 (-6.40,0.19) | 0.01 (-4.15,4.18) | -4.00 (-8.30,0.31) | -2.43 (-6.68,1.82) | -2.99 (-7.03,1.05) |
|  | 2019 (N=135) | -1.32 (-4.55,1.92) | -0.35 (-4.44,3.75) | -1.16 (-5.39,3.07) | 1.81 (-2.37,5.98) | -0.73 (-4.70,3.23) |
| Schools |  |  |  |  |  |  |
|  | Other schools (N=18) | Reference | Reference | Reference | Reference | Reference |
|  | School of Basic Medicine (N=173) | 0.72 (-2.44,3.89) | -0.83 (-4.84,3.17) | 1.93 (-2.20,6.07) | -2.71 (-6.79,1.38) | -2.65 (-6.53,1.23) |
|  | School of Pharmacy (N=112) | 1.50 (-1.97,4.96) | **5.83 (1.46,10.21)** | **5.05 (0.52,9.58)** | 2.49 (-1.97,6.96) | 2.43 (-1.82,6.67) |
|  | School of Public Health (N=69) | 5.98 (-0.10,12.06) | 5.57 (-2.11,13.26) | 5.20 (-2.75,13.14) | 2.68 (-5.16,10.52) | 3.85 (-3.60,11.30) |
| Have you served as a TA this semester? | |  |  |  |  |  |
|  | No (N=198) | Reference | Reference | Reference | Reference | Reference |
|  | Yes (N=174) | 0.92 (-1.83,3.66) | -1.62 (-5.08,1.85) | 0.67 (-2.91,4.25) | 1.38 (-2.16,4.92) | **-3.42 (-6.78, -0.06)** |
| Are you attending your first TA training | |  |  |  |  |  |
|  | No (N=9) | Reference | Reference | Reference | Reference | Reference |
|  | Yes (N=363) | 7.19 (-0.95,15.33) | -4.52 (-14.80,5.77) | -4.15 (-14.78,6.48) | -2.96 (-13.45,7.54) | 1.01 (-8.96,10.98) |
| Want to be a college teacher? | |  |  |  |  |  |
|  | Neutral (N=116) | Reference | Reference | Reference | Reference | Reference |
|  | No (N=19) | **6.89 (0.94,12.83)** | 0.95 (-6.56,8.46) | 3.53 (-4.23,11.29) | 6.12 (-1.55,13.78) | 1.31 (-5.97,8.59) |
|  | Yes (N=237) | 3.86 (-1.93,9.65) | -1.98 (-9.30,5.34) | -0.32 (-7.89,7.24) | 5.22 (-2.25,12.69) | 0.74 (-6.36,7.84) |
| Dose training improve your confidence? | |  |  |  |  |  |
|  | No (N=21) | Reference | Reference | Reference | Reference | Reference |
|  | Unsure (N=85) | -0.13 (-5.68,5.41) | 0.15 (-6.85,7.16) | -2.90 (-10.15,4.34) | -1.50 (-8.65,5.65) | -2.14 (-8.93,4.65) |
|  | Yes (N=266) | -1.66 (-7.52,4.20) | -3.29(-10.69,4.12) | -5.19 (-12.85,2.46) | -0.53 (-8.09,7.03) | -4.39 (-11.57,2.79) |

**Supplementary table 4 Multivariable-adjusted estimates for confidence level of question 6 to 10 before and after training**

| **Variables** | | **β(95CI%) for Q6** | **β(95CI%) for Q7** | **β(95CI%) for Q8** | **β(95CI%) for Q9** | **β(95CI%) for Q10** |
| --- | --- | --- | --- | --- | --- | --- |
| Year |  |  |  |  |  |  |
|  | 2017 (N=107) | Reference | Reference | Reference | Reference | Reference |
|  | 2018 (N=130) | -2.85 (-5.69, -0.01) | -1.55 (-4.98,1.87) | -2.16 (-6.36,2.03) | -1.00 (-4.61,2.61) | -1.16 (-4.29,1.98) |
|  | 2019 (N=135) | -2.41 (-5.20,0.38) | -1.67 (-5.04,1.69) | 1.43 (-2.68,5.55) | -0.90 (-4.45,2.65) | 0.24 (-2.84,3.32) |
| Schools |  |  |  |  |  |  |
|  | Other schools (N=18) | Reference | Reference | Reference | Reference | Reference |
|  | School of Basic Medicine (N=173) | -0.98 (-3.71,1.75) | -0.86 (-4.15,2.43) | -1.54 (-5.57,2.48) | -1.12 (-4.59,2.35) | -1.94 (-4.95,1.07) |
|  | School of Pharmacy (N=112) | -1.58 (-4.57,1.41) | 2.63 (-0.97,6.24) | 3.38 (-1.02,7.79) | 3.87 (0.07,7.67) | 1.68 (-1.62,4.97) |
|  | School of Public Health (N=69) | -2.32 (-7.56,2.92) | 2.55 (-3.77,8.88) | 4.15 (-3.58,11.88) | 1.78 (-4.88,8.45) | 2.86 (-2.92,8.64) |
| Have you served as a TA this semester? | |  |  |  |  |  |
|  | No (N=198) | Reference | Reference | Reference | Reference | Reference |
|  | Yes (N=174) | 0.18 (-2.18,2.55) | 1.16 (-1.69,4.02) | 3.07 (-0.42,6.55) | 2.38 (-0.63,5.39) | 0.25 (-2.36,2.86) |
| Are you attending your first TA training | |  |  |  |  |  |
|  | No (N=9) | Reference | Reference | Reference | Reference | Reference |
|  | Yes (N=363) | -1.00 (-8.01,6.01) | 5.20 (-3.27,13.66) | 2.51 (-7.83,12.86) | -1.92 (-10.84,7.00) | 1.87 (-5.87,9.61) |
| Want to be a college teacher? | |  |  |  |  |  |
|  | Neutral (N=116) | Reference | Reference | Reference | Reference | Reference |
|  | No (N=19) | -1.04 (-6.16,4.08) | 1.02 (-5.16,7.20) | 1.29 (-6.27,8.85) | 4.87 (-1.65,11.39) | 2.56 (-3.09,8.21) |
|  | Yes (N=237) | -0.96 (-5.95,4.03) | -2.05 (-8.07,3.97) | -0.77 (-8.13,6.60) | 3.60 (-2.75,9.95) | 2.64 (-2.87,8.15) |
| Dose training improve your confidence? | |  |  |  |  |  |
|  | No (N=21) | Reference | Reference | Reference | Reference | Reference |
|  | Unsure (N=85) | -1.10 (-5.87,3.68) | 1.00 (-4.77,6.76) | -0.20 (-7.25,6.85) | -2.12 (-8.20,3.96) | -2.20 (-7.47,3.08) |
|  | Yes (N=266) | -2.69 (-7.74,2.36) | 0.75 (-5.34,6.85) | -1.85 (-9.30,5.60) | -3.77 (-10.19,2.66) | -2.28 (-7.86,3.29) |
